# Study of Thermal Effects in Fused-Tapered Pure Passive Fibers and Signal Combiners

**DOI:** 10.3390/nano15010062

**Published:** 2025-01-02

**Authors:** Yuyi Yin, Tingwu Ge, Guanrui Zhao, Ruoyu Jia, Zhiyong Wang

**Affiliations:** School of Physics and Optoelectronic Engineering, Beijing University of Technology, Beijing 100124, China; yinyuyi@emails.bjut.edu.cn (Y.Y.); getingwu@bjut.edu.cn (T.G.); S202278077@emails.bjut.edu.cn (G.Z.); S202378091@emails.bjut.edu.cn (R.J.)

**Keywords:** thermal effects, fused-tapered, high-power fiber laser, optical fiber combiner, thermal management

## Abstract

This paper investigates the thermal effects in fused-tapered passive optical fibers under near-infrared absorption. The thermal effect is primarily caused by impurities, such as OH-, which absorb incident light and generate heat. Using the finite element method, the volume changes during fiber tapering were simulated, influencing power density and thermal distribution. The heat conduction equation and ray optics were employed to analyze the thermal distribution in tapered fibers and signal combiners. Results show that at 5 kW power, the temperature peak for a single fiber reaches 316.73 °C, while for bundled fibers, the temperature rises significantly as the bundle configuration increases from 7 × 1 to 61 × 1, peaking at 453.09 °C—an increase of 171.6%. Variations in tapering ratio and length also notably affect the thermal behavior. Increasing the tapering ratio from 5 to 8 results in a 52.5% temperature rise, while doubling the taper length from 25 mm to 50 mm reduces the temperature peak by 59.1%. These findings offer important insights for the design and optimization of high-power optical fiber combiners and their heat dissipation structures.

## 1. Introduction

With the advancement of high-power fiber laser technologies, managing thermal effects in fiber transmission systems has become a critical challenge. Tapering technique is crucial for optical fiber post-processing, as it significantly influences waveguide geometry and thermal characteristics [1]. Fused-tapered passive optical fibers and combiners, owing to their unique structures, exhibit pronounced thermal effects under high-power conditions [2]. Such thermal issues impact not only the optical transmission performance but also the operational reliability of fiber lasers.

In recent years, researchers have gradually realized that impurities in optical fibers, especially OH- ions, will produce significant thermal effects after absorbing near-infrared light waves [3]. The generation mechanism and distribution characteristics of this thermal effect are critical to optimizing fiber design and improving system performance. For example, Chen et al. (2020) reported an all-fiber Raman fiber amplifier with an output power exceeding 2 kW, achieving a high conversion efficiency of 79.35% at 1130 nm, and obtaining a good beam quality with M^2^ of 2.8 [4]. Yan et al.’s (2022) study showed that the self-imaging effect based on square core fibers can be used to construct an all-fiber coherent beam synthesis source [5]. The influence of various parameters on beam quality (M^2^) and efficiency was studied through numerical simulation, proving that a laser beam close to the diffraction limit can be achieved, which helps to reduce the impact of thermal effects on beam quality [6]. Lou et al. (2023) directly targeted the problem of optical fiber thermal effects and studied the transmission mode [4], laser-induced damage threshold (LIDT), temperature distribution and thermal stress distribution of optical fiber through the finite element method (FEM). Studies have shown that under laser irradiation, the center of the front surface of the optical fiber is affected by severe thermal effects and is prone to damage [7]. Gao Huaxing (2023) constructed a three-dimensional transient thermal conduction equation for laser crystals [8]. Based on the input power, heat transfer coefficient, pump mode, and fiber parameter values, MATLAB software (R2023B) was used to simulate the thermal effects of high-power fiber lasers. The results show that increasing the heat transfer coefficient can reduce the fiber temperature. The larger the input power, the higher the laser temperature. The larger the fiber radius, the lower the core temperature, but spectrum broadening will occur [9]. However, due to the special geometric structure and complex optical properties of tapered optical fibers, traditional thermal effect analysis methods cannot provide accurate descriptions of the heat distribution.

In addition, as the power of fiber lasers continues to increase, the thermal management issues of fiber signal combiners [10,11], as key components, have become increasingly important. Currently, scholars are conducting research on fiber optic signal combiners. For example, Huang Jianbin (2021) achieved flat-top light output by performing special processing on the cone area of the optical fiber signal combiner and the output fiber, and twisting the output fiber. A section of 200/220/360 μm optical fiber forms the transition between the cone area and the output fiber. The resulting 4 × 1 signal combiner has a uniform intensity distribution within the beam waist of 4.88 mm, showing a flat-top distribution. Through calculation, it can be seen that the beam flatness within this range is below 0.1, and the signal power that the optical fiber signal combiner can withstand is above 2 kW [8]. Wang Jiawei (2024) developed a (6 + 1) × 1 reverse pump/signal combiner with high pump optical coupling efficiency and high beam quality maintenance with both input and output using 50 μm large core diameter signal fiber [12]. Simulation software (6.0 version) was used to analyze the effects of the length of the taper area, the tapering ratio and the refractive index of the glass tube on the pump light coupling efficiency, and the effect of the fiber core axial offset on the signal light transmission efficiency and beam quality [13]. These studies show that fiber signal combiners play a key role in high-power fiber laser systems, but their thermal effect distribution rules are still unclear. Current research focuses on the analysis of the thermal effects of a single optical fiber or a single cone area. In-depth research is needed on the thermal superposition effect of multiple optical fibers in the combiner and the impact of different combining parameters on thermal distribution. The solution to these problems is not only related to the performance and life of the combiner, but also directly affects the reliability and stability of the entire high-power optical fiber system.

Therefore, in-depth research on the distribution of thermal effects in fused-tapered pure passive optical fibers and signal combiners and exploration of key influencing factors are of great significance for promoting the development of high-power fiber laser technology. This study not only helps to optimize the design of optical fibers and combiners, but also provides a theoretical basis and practical guidance for solving the heat dissipation problem of high-power optical fiber systems. The innovation of this article is in the following: First, the thermal distribution characteristics of fused-tapered optical fibers and signal combiners under different input power conditions are systematically studied, focusing on the analysis of the thermal superposition effect in multi-fiber combiners; second, the finite element method and ray optics model are combined to accurately analyze the thermal effect distribution and heat conduction mechanism of tapered optical fibers; third, the influence of parameters such as the number of optical fibers, taper ratio and taper length on thermal distribution is quantitatively analyzed, providing a theoretical basis for the design of optical fiber signal combiners. The research results not only help to optimize the design of high-power fiber laser systems, but also provide new ideas and technical support for heat dissipation problems under high-power conditions, and promote the further development of fiber laser technology in the fields of national defense, communications and industrial processing, which has important theoretical and practical significance.

## 2. Theory and Model of Thermal Effect of Fused-Tapered Optical Fiber

**Figure 1:**
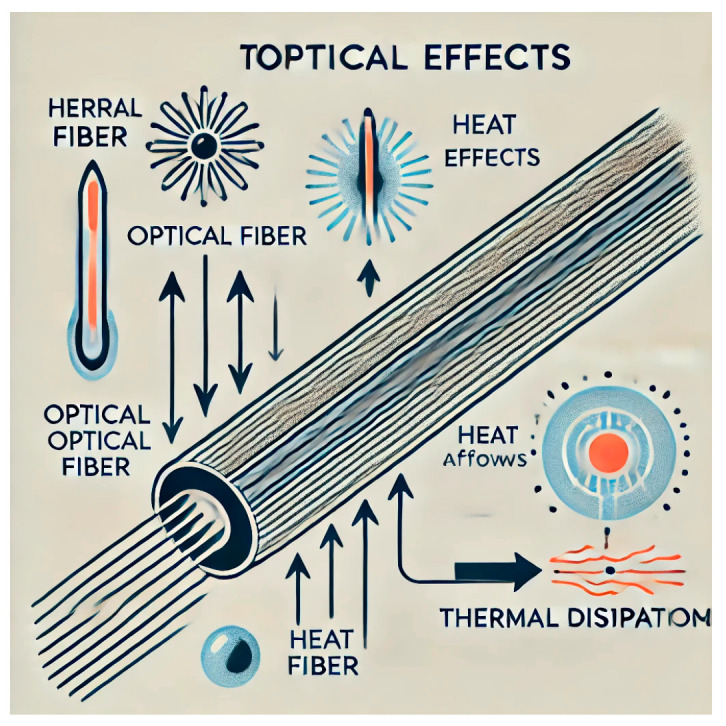
Thermal effect model in tapered optical fiber region.

This section presents the theoretical foundation and modeling approach for analyzing thermal effects in fused-tapered optical fibers. The mechanisms of heat generation, thermal loss, and distribution are discussed, followed by the development of a numerical model to simulate temperature fields. The schematic diagram of this study is shown in Figure 1. The schematic highlights the tapered geometry with different cross-sectional areas, the heat generation zone caused by impurity absorption, axial heat conduction, and radial heat dissipation.

### 2.1. Mechanisms of Heat Generation in Optical Fibers

The thermal effect in fused-tapered fibers is mainly due to the absorption of impurities and the structural change caused by the tapering process. Hydroxyl ions (OH^−^) absorb near-infrared photons and convert their energy into heat [7,9], resulting in local temperature increases [13,14,15,16,17]. The tapering process increases the power density by reducing the cross-sectional area of the fiber [18,19], further exacerbating the thermal effects.

In order to accurately measure the thermal effect caused by OH^−^ absorption of incident light waves, research work should be carried out from the perspectives of classical electromagnetic theory and quantum theory of light to deeply explore the absorption mechanism of OH^−^ groups on light waves in quartz optical fibers [20,21].

Based on Maxwell’s equations, the relationship between frequency and wave vector can be derived:

(1) $$\frac{kc}{\omega }=n+i\kappa$$

where *k* is the incident light wave vector, *n* is the refractive index, and κ is the extinction coefficient (related to the absorption of light). These satisfy the following relationship:

(2) $$\frac{kc}{\omega }=n+i\kappa$$

where $\chi^{\prime }$ and $\chi^{\prime \prime }$ are the real and imaginary parts of the electric susceptibility, respectively, which are related to the transmission and absorption of light.

In the field of quantum optics, light waves are considered to be statistical waves formed by the random vibration of many photons. The absorption process of light by microscopic particles is manifested as follows:

(3) $$\bar{I\left(z\right)}=\bar{I_{0}}e^{\frac{2\omega Kz}{c}}\;$$

where *z* is the transmission distance. Based on the power balance condition in microscopic theory and the quantum mechanical derivation of the Einstein coefficient [22], the absorption coefficient *K* is expressed as the following:

(4) $$K\left(\omega \right)=\frac{NB_{12}\hbar \omega_{0}}{ncV}F_{L}\left(\gamma ,\omega \right)$$

where *N* is the number of atoms, *V* is the volume of space, *n* is the refractive index of the material, $\omega_{0}$ is the transition frequency, and $B_{12}$ is the stimulated radiation coefficient. $F_{L}\left(\gamma ,\omega \right)$ is the Lorentzian linear function [23], which reflects the relationship between the absorption coefficient and the frequency of the absorbed light wave:

(5) $$F_{L}\left(\gamma ,\omega \right)=\frac{\frac{\gamma }{\pi }}{{\left(\omega -\omega_{0}\right)}^{2}+\gamma^{2}}$$

The final form of the absorption coefficient is the following:

(6) $$K\left(\omega \right)=\left(\frac{2Nn^{2}V}{3\pi \epsilon_{0}g_{1}c^{2}}\right)\left(\frac{e^{2}{\left|D_{12}\right|}^{2}}{\hbar \omega_{0}^{2}}\right)F_{L}\left(\gamma ,\omega \right)$$

According to the above formula, it can be observed that in a specific space, the absorption coefficient of the same type of particles with the same number is only related to the electric polarization behavior of the particles in the corresponding dielectric and the frequency of the light waves absorbed by the particles. In addition, the relationship between the absorption coefficient and the frequency of the light wave presents a Lorentz line shape. By introducing parameters related to the absorption of light waves by OH^−^ groups, the absorption coefficient of OH^−^ to light waves in quartz glass can be determined. Based on this, the power loss caused by the absorption of incident light by OH^−^ groups can be quantitatively evaluated. Furthermore, using the corresponding heat conversion coefficient, the heat power generated by the absorption of incident light by OH^−^ groups can be quantitatively calculated.

### 2.2. Thermal Effect Model of Cone Zone

Considering the influence of fiber volume changes during the taper process on power density and thermal effects, a thermal effect model in the taper region is established. The temperature field of the tapered region is controlled by the heat conduction equation in the cylindrical coordinate system:

(7) $$\rho c\frac{\partial T}{\partial t}=\frac{1}{r}\frac{\partial T}{\partial r}\left(kr\frac{\partial T}{\partial r}\right)+\left(k\frac{\partial T}{\partial z}\right)+Q\left(r,z\right)$$

where ρ and *c* are the density and specific heat capacity of the quartz fiber, respectively, and *k* is the thermal conductivity. *Q* represents the heat source, which refers to the heat energy generated per unit volume per unit time. In the model, *Q* is a variable related to the OH^−^ concentration.

During the tapering process, the geometric shape function of the tapered area of the combiner is closely related to parameters such as the temperature, duration, and initial length of the tapered area, while the function needs to maintain a constant volume. The ideal tapering operation should achieve precise point-to-point matching, which means that the small segments of the tapered area curve should approach zero. It is assumed that the fiber taper process is a uniform taper, that is, each position of the fiber moves and changes with the tapering process. In order to describe the change in fiber volume, a partial differential equation is established to constrain volume conservation. The change in the cross-sectional area of the fiber with the axial position Z is expressed as follows:

(8) $$A\left(Z\right)=\pi r{\left(Z\right)}^{2}$$

where $r\left(Z\right)$ is the radius of the fiber that varies with axial position.

According to the principle of volume conservation, the volume of the optical fiber remains unchanged before and after tapering. The volume change of the optical fiber is calculated by the volume change rate formula. The volume change rate *V* formula is the following:

(9) $$V=\int_{Z=0}^{L}\pi r{\left(Z\right)}^{2}dZ$$

where $r\left(Z\right)$ is the radius of the fiber varying with axial position, and *L* is the total tapered length of the fiber.

According to the definition of the optical fiber loss coefficient, after the laser is transmitted through an optical fiber for any distance [24], the relationship between the input and output power is as follows:

(10) $$P_{in}=P_{0}e^{-\alpha L^{\prime }}$$

where α is the fiber loss coefficient, $L^{\prime }$ is the transmission distance, and a small fiber element is taken axially in the cone area. The power loss of the incident light passing through this small fiber element is as follows:

(11) $$P_{loss}=P_{0}e^{-\alpha L^{\prime }}-P_{0}e^{-\alpha \left(l+dl\right)}$$

where *l* is the distance coordinate of the transmission direction of the incident light.

The thermal effect of the optical fiber is closely related to the heat loss power of the optical fiber [25]. The heat loss power of an optical fiber $P_{heat}$ is as follows:

(12) $$P_{heat}=V\times P_{loss}\times \alpha_{{\mathrm{OH}}^{-}}\times \eta_{heat}$$

where $\alpha_{{\mathrm{OH}}^{-}}$ is the absorption coefficient of OH^−^, and $\eta_{heat}$ is the heat conversion efficiency. This formula simulates the heat loss process of an optical fiber by its absorption of light energy and converting it into heat energy through impurities in the optical fiber.

### 2.3. Thermal Effect Model of Cone Zone

The heat conduction equation is used to describe the diffusion process of heat in the optical fiber due to temperature differences. The following heat conduction equation can be used to model heat distribution in the optical fiber system:

(13) $$\frac{\partial T}{\partial t}=\alpha \nabla^{2}T$$

where *T* is temperature, *t* is time, α is thermal diffusivity, and $\nabla^{2}$ is the Laplace operator, which represents the diffusion of heat in space. The heat conduction equation assumes that heat is transferred in the optical fiber material by conduction and that the thermal properties of the material are uniform. By solving this equation, the temperature distribution in the optical fiber can be obtained, and especially the heat generated by local light absorption can be modeled. In actual numerical simulations, considering the geometry of the optical fiber (such as fiber reduction and fiber bundle configuration) and the variation of power density at different locations, we combine the heat conduction equation with boundary conditions for the numerical solution. This helps to obtain the temperature distribution in the optical fiber and its connection area (such as the reduction end).

Under steady state conditions, the heat conduction equation can be simplified as follows:

(14) $$0=\frac{1}{r}\frac{\partial }{\partial r}\left(kr\frac{\partial T}{\partial r}\right)+\frac{\partial }{\partial z}\left(k\frac{\partial T}{\partial z}\right)+Q\left(r,z\right)$$

For axisymmetric problems in cylindrical coordinates, the temperature distribution varies mainly along the axial *z* direction. Considering uniformity in the *r* direction, the one-dimensional problem can be further simplified:

(15) $$k\frac{d^{2}T}{dz^{2}}+Q\left(z\right)=0$$

Assuming that the heat source $Q\left(z\right)$ is related to the position *z*, this differential equation can be further solved. For constant thermal conductivity *k* and uniform heat source *Q*, the analytical solution is the following:

(16) $$T\left(z\right)=-\frac{Q}{2k}z^{2}+C_{1}z+C_{2}$$

where $C_{1}$ and $C_{2}$ are unknown integral constants, which are determined according to the boundary conditions.

We set the temperature of the fiber at *z* = 0 to $T_{0}$ and the temperature at the end of the fiber at *z = L* to $T_{L}$, and we obtain $T_{0}=C_{2}$.

(17) $$T_{L}=-\frac{Q}{2k}L^{2}+C_{1}L+T_{0}$$

So, $C_{1}=\frac{T_{L}-T_{0}+\frac{Q}{2k}L^{2}}{L}$. The final temperature distribution expression is the following:

(18) $$T\left(z\right)=-\frac{Q}{2k}z^{2}+\left(\frac{T_{L}-T_{0}+\frac{Q}{2k}L^{2}}{L}\right)z+T_{0}$$

The heat conduction equation describes how the absorbed light energy diffuses in the optical fiber and how the heat is transferred to other areas by heat conduction. By solving the heat conduction equation, we can obtain the temperature distribution at each location in the optical fiber, especially in the reduced-diameter part of the optical fiber and the combiner area. The temperature distribution is crucial to the performance and reliability of the equipment.

**Figure 2:**
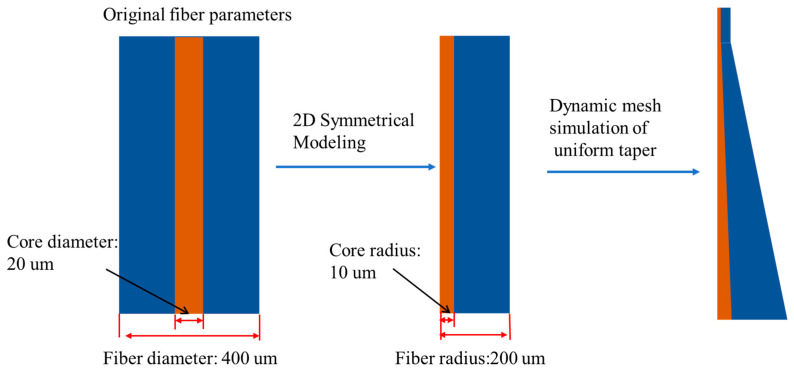
Schematic diagram of the composition of the thermal effect model in the cone region.

In order to reduce computational complexity and improve simulation efficiency, symmetry and geometric approximation methods are often used to simplify complex structures. Since the geometric shape and physical properties of the optical fiber tapering process are rotationally symmetrical, the amount of calculation can be significantly reduced by simplifying it into a two-dimensional symmetric model while ensuring the accuracy and reliability of the simulation results. The original fiber diameter is set to 400 μm, the core diameter is set to 20 μm, and a two-dimensional distribution signal symmetric model is used to simplify the calculation. Assuming that the object has rotational symmetry along a certain axis, only the cross-section needs to be analyzed, thereby reducing the amount of calculation. The material deformation during the taper process is simulated by setting the geometric parameters of the cylindrical section and applying dynamic mesh technology. The model is simplified to a two-dimensional structure with rotational symmetry around the central axis. By setting key parameters such as the initial radius, the length after stretching, and the radius of the cone top, dynamic simulation is performed to simulate the stress distribution and geometric shape changes during stretching and deformation [26]. The finite element method is used to simulate the volume change during the fiber taper process. The shape of the cone area is close to that of a standard truncated cone. In order to simplify the simulation model, a truncated cone model is used in the cone area of the combiner in the simulation. The specific process is shown in Figure 2.

The relevant parameters of the cone zone thermal effect model are shown in Table 1.

**Figure 3:**
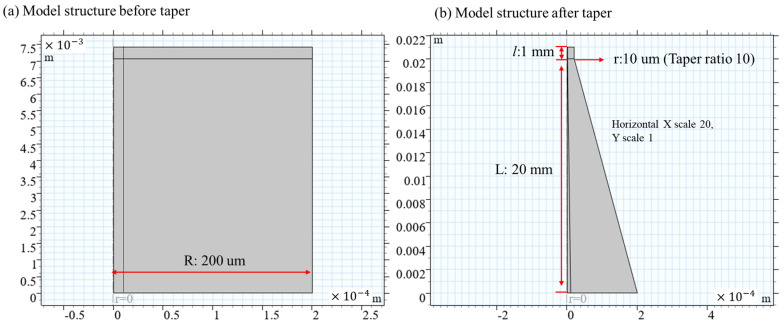
Dynamic mesh pulling cone process.

Two-dimensional axisymmetric modeling is adopted, and the dynamic mesh taper process is shown in Figure 3.

**Figure 4:**
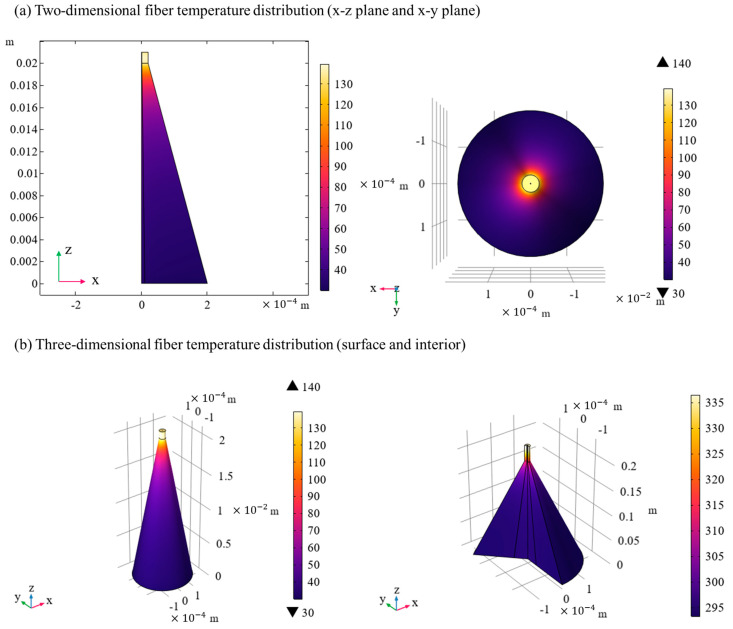
Tapered fiber temperature distribution.

Through the above steps, the model obtains the power density and heat loss power distribution at different points in the optical fiber during the tapering process. Combined with the heat conduction equation, the model further calculated the temperature distribution of the tapered optical fiber and obtained the thermal field in the steady state [26], as shown in Figure 4. The simulation can reflect the thermal effect of an optical fiber under high power conditions, especially at the output end of a tapered optical fiber, where the temperature gradient is significant.

By constructing a heat source distribution model based on the heat source function expression, the distribution difference of OH^−^ concentration in different areas of the beam combiner cone can be more clearly identified. The cone heat source distribution model obtained by simulation is consistent with the theoretical analysis results, and both conform to the mass conservation principle and Fick’s second law of thermodynamics.

**Figure 5:**
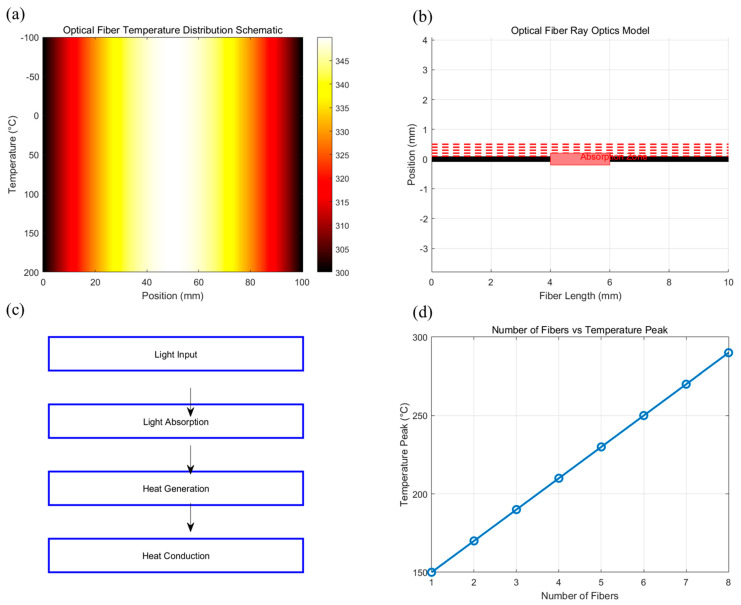
Simulation of thermal effects and optical propagation in fused-tapered optical fibers. (**a**) Heat conduction equation schematic; (**b**) ray optics model schematic; (**c**) combined simulation process flowchart; (**d**) parameter sensitivity analysis chart.

As shown in Figure 5, the key processes involved in the thermal and optical behavior of a fused-tapered fiber under high-power conditions are demonstrated. The heat conduction equation explains how the temperature gradient develops along the fiber, while the ray optics model shows how the light interacts with impurities, resulting in localized heating. A combined simulation flow chart then ties these models together, showing the sequential steps involved in the simulation process. Finally, parameter sensitivity analysis plots reveal how different design parameters affect the thermal performance. Together, these plots form a solid foundation for understanding the complex thermal effects in tapered fibers, and the insights gained from these simulations are critical for optimizing fiber design and ensuring effective heat dissipation in high-power applications.

### 2.4. Finite Element Simulation and Model Verification

In finite element simulation, the computational cost of each simulation mainly depends on the complexity of the computational domain and the number of iterations of the selected solver. A single simulation takes about 2.5–4 h and consumes about 3.5–6 GB of memory. To ensure the stability and credibility of the results, multiple groups of simulations were performed for different input powers (1 kW, 2 kW, 3 kW, 4 kW, 5 kW), taper ratios (5, 6, 7, 8, 10, 15), taper lengths (25 mm, 30 mm, 35 mm, 40 mm, 45 mm, 50 mm) and the number of combined optical fibers (7 × 1, 19 × 1, 37 × 1, 61 × 1). Each group of simulations was repeated three times to verify the repeatability of the results, and a total of 57 simulations were completed. The specific computational cost and parameter settings are shown in Table 2.

Through the above simulations, detailed power density, heat loss power distribution, temperature field variation law and thermal effect characteristics of the combiner were obtained, which provided an important basis for optimizing optical fiber design and ensuring the stability of high-power optical fiber systems.

## 3. Results and Discussion

The thermal effects in single pure passive optical fibers were analyzed under varying power levels, taper ratios, and taper lengths. The study highlights how these parameters influence the axial temperature gradient and heat source distribution, with the highest temperatures consistently observed at the tapered output end.

### 3.1. Single Pure Passive Optical Fiber

#### 3.1.1. Effect of Power

**Figure 6:**
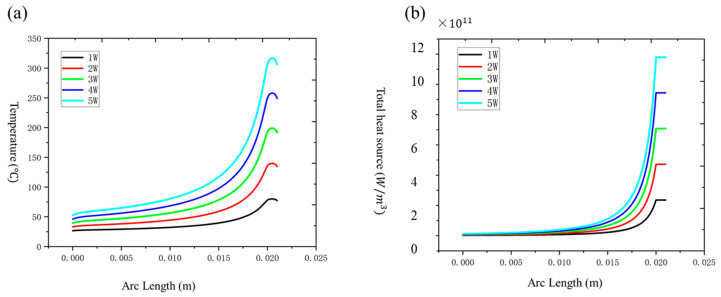
(**a**) Impact of power on axial temperature distribution in a single passive fiber; (**b**) heat source distribution of a single pure passive fiber at different powers.

As the power increased from 1 kW to 5 kW, the temperature at the tapered output end rose significantly, with the peak value escalating from 80 °C at 1 kW to 316.73 °C at 5 kW. The temperature gradient became more pronounced along the axial direction as power increased, indicating the emergence of localized heat accumulation at higher power levels, as shown in Figure 6a. The corresponding heat source intensity also increased linearly, from $2.36\times 10^{10}\;W/m^{3}$ at 1 kW to $11.8\times 10^{10}\;W/m^{3}$, reflecting enhanced thermal effects due to higher power densities, as shown in Figure 6b. These results underscore the need for effective cooling mechanisms to maintain thermal stability under high-power conditions [27].

#### 3.1.2. Effect of Taper Ratio

**Figure 7:**
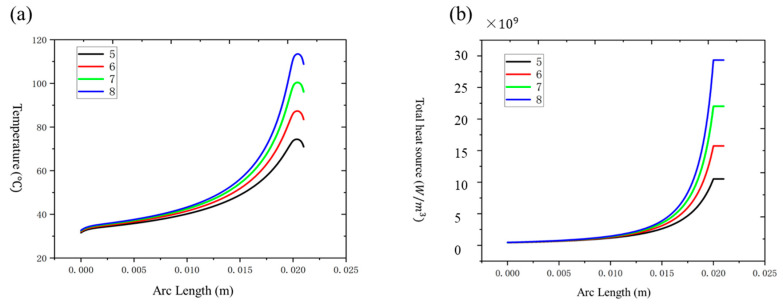
(**a**) Temperature distribution of a single pure passive fiber with different draw-taper ratio; (**b**) heat source distribution of a single pure passive fiber with different pull-taper ratios.

The taper ratio, representing the diameter change of the optical fiber during tapering, had a substantial impact on the thermal behavior [28]. When the taper ratio increased from 5 to 8, the maximum temperature at the tapered output end rose from 74.37 °C to 113.46 °C. Higher taper ratios intensified the thermal effect by concentrating power density in the tapered region, leading to significant local heat accumulation, as shown in Figure 7a,b. This observation highlights the importance of optimizing the taper ratio to balance the thermal load and optical transmission efficiency in fiber systems.

In terms of heat source distribution, the heat source intensity of a single pure passive fiber under different taper ratios (5, 6, 7, 8) also shows an upward trend as the taper ratio increases. When the taper ratio is 5, the heat source intensity is approximately 10.50 × 10^9^ W/m^3^. As the taper ratio increases to 8, the heat source intensity of a single pure passive optical fiber increases significantly to 2.94 × 10^10^ W/m^3^. Although the heat source distribution is generally gentle, a higher taper ratio will enhance the heat source intensity inside the optical fiber, especially the end area, which exhibits a strong heat accumulation effect.

Taken together, as the taper ratio increases, the temperature and heat source intensity inside a single pure passive optical fiber increase significantly, and the temperature gradient is noticeably distributed along the axial direction. Especially at the tapered output end, the temperature and heat source intensity reach the maximum value. This phenomenon shows that a high draw-to-cone ratio not only increases the thermal effect of the optical fiber, but also makes the local area at the end of the optical fiber a key point for heat concentration. This enhancement of thermal effects needs to be carefully controlled in practical applications to avoid material or structural problems caused by excessive heat accumulation.

#### 3.1.3. Effect of Taper Length

**Figure 8:**
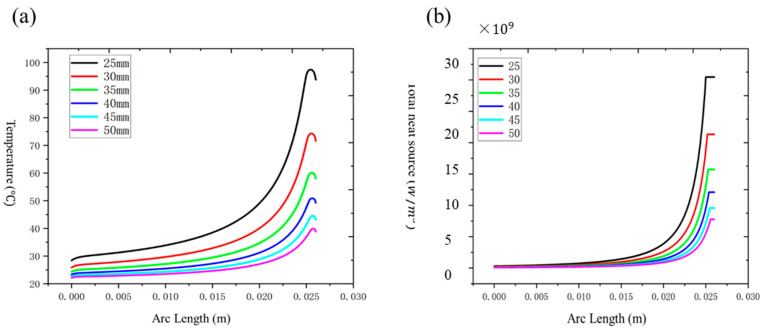
(**a**) Temperature distribution of a single pure passive fiber with different pulling cone lengths; (**b**) heat source distribution of a single pure passive fiber with different pulling cone lengths.

The taper length influenced both the temperature distribution and heat source intensity within the fiber. Shorter taper lengths (25 mm) resulted in higher peak temperatures (97.41 °C), with heat concentrated in a localized region. Conversely, longer taper lengths (50 mm) dispersed the heat more evenly, reducing the peak temperature to 39.87 °C and minimizing localized overheating, as shown in Figure 8a,b. This trend demonstrates that extending the taper length is an effective strategy for enhancing thermal stability and uniformity in optical fibers.

By analyzing the temperature and heat source distribution of a single pure passive optical fiber at different taper lengths, as shown in Figure 8, it can be seen that with the increase of the taper length, the temperature distribution of the optical fiber shows a significant change. At shorter taper lengths (25 mm and 30 mm), the temperature of the optical fiber is higher, especially concentrated in a certain section of the optical fiber, and the local temperature is more obvious, resulting in a higher temperature peak of 97.41 °C and 74.28 °C, respectively. As the taper length increases, the temperature of a single pure passive optical fiber gradually decreases. When the taper length increases to 50 mm, the temperature peak of the optical fiber decreases to 39.87 °C. This shows that a longer taper length helps to evenly distribute heat and reduce local overheating. It can be seen from the temperature distribution diagram that the temperature gradient of a single pure passive optical fiber is mainly distributed along the axial direction of the optical fiber, especially at the output end of the cone, where the temperature is higher, indicating that the heat accumulation is most distinct here. A shorter taper length will cause the temperature to concentrate at a certain location, while a longer taper length will help to disperse the heat more evenly and reduce local temperature differences.

It can be seen from Figure 8b that as the tapered length increases, the heat source intensity inside a single pure passive optical fiber shows a gradually decreasing trend. Specifically, when the tapered length is 25 mm, the heat source intensity of a single pure passive optical fiber is $3.05\times 10^{10}\;W/m^{3}$; when the tapered length is 35 mm, the heat source intensity of a single pure passive optical fiber is 1.57 × 10^10^ W/m^3^; and when the tapered length is 50 mm, the heat source intensity of a single pure passive optical fiber is $7.77\times 10^{9}\;W/m^{3}$. This shows that extending the tapered length can effectively reduce the heat accumulation inside the optical fiber, reduce local overheating, and thereby improve the thermal stability of the optical fiber. The increase in the tapered length not only reduces the temperature peak, but also significantly reduces the heat source intensity, making the heat source distribution more uniform. Especially at longer taper lengths (such as 45 mm and 50 mm), the heat source distribution curve of a single pure passive fiber is flatter, indicating that the heat source is distributed more evenly at this time, effectively avoiding excessive local heat concentration, thereby reducing temperature differences in optical fibers.

In summary, as the taper length increases, the heat distribution of a single pure passive optical fiber becomes more uniform, and the temperature and heat source intensity gradually decrease. This phenomenon shows that appropriately increasing the taper length can effectively reduce the temperature peak of the optical fiber and reduce the heat source intensity, which is beneficial to the thermal stability and long-term safety of the optical fiber.

### 3.2. Multiple Passive Optical Fibers Combined

Compared with a single optical fiber, a combined bundle of multiple pure passive optical fibers shows significant differences in temperature distribution, spatial distribution of heat sources and heat dissipation effect. When multiple optical fibers are combined, due to the heat conduction between optical fibers, the local temperature peak is higher and the temperature distribution is more complex; especially at the junction of optical fibers, heat accumulation is prone to occur [28]. The heat source is also more unevenly distributed among multiple optical fibers, making heat dissipation more difficult and the temperature gradient larger, affecting the thermal stability and performance of the optical fiber. Analyzing the temperature distribution under different parameters helps ensure that each fiber can maintain a stable working condition despite the bundling process and will not cause increased losses or equipment failure due to local overheating. To this end, we set the power to 2 KW, the tapering ratio to 10, and the tapering length to 20 mm. We analyzed the fiber temperature and heat source distribution under different bundle numbers (7 × 1, 19 × 1, 37 × 1, 61 × 1).

The wall thickness of the casing is equal to the combined beam diameter. The specific corresponding thickness is shown in Table 3. As the number of combining layers increases, the number of optical fibers, the number of diagonals, the diagonal length, and the diameter of the casing all show a gradually increasing trend, and the combining structure becomes more complex.

**Figure 9:**
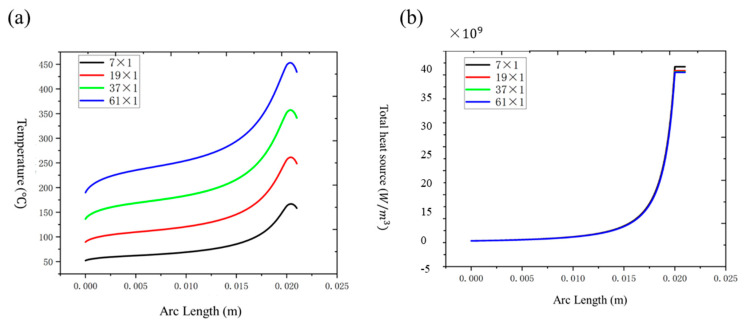
(**a**) Temperature distribution within an optical fiber with different numbers of bunched roots; (**b**) distribution of heat sources within an optical fiber with different numbers of bunched roots.

The fiber temperature and heat source distribution under different bundling numbers (7 × 1, 19 × 1, 37 × 1, 61 × 1) are shown in Figure 9a,b.

It can be seen from Figure 9a,b that as the number of combined bundles increases, the temperature peak of the optical fiber shows an obvious upward trend. As the number of bundled fibers increases from 7 × 1 to 61 × 1, the temperature peak value of the optical fiber gradually increases. Specifically, under 7 × 1 bundling, the optical fiber temperature peak value is 166.78 °C; at 19 × 1, the temperature peak rose to 261.36 °C; it further increased to 357.18 °C at 37 × 1, and finally reached the highest peak of 453.09 °C at 61 × 1. This trend shows that as the number of combined fibers increases, the heat concentration inside the fiber bundle becomes more significant. The temperature gradient in the cone area of the combiner is mainly distributed along the axial direction. The heat transfer process in the entire cone area can be regarded as the gradual transfer of heat from the output end of the cone area to the input end, and at the same time, heat is transferred from the surface of the cone area to the central area. In addition, the overall temperature of the cone zone increases with heat generation from the internal heat sources.

However, although the temperature peak increases significantly with the increase in the number of bundled fibers, the total heat source density of the optical fiber remains relatively stable. At 7 × 1, the maximum heat source intensity is 3.67 × 10¹⁰ W/m^3^, and when the number of bundled fibers increases to 19 × 1, the heat source density drops slightly to 3.59 × 10¹⁰ W/m^3^. When the number of bundled fibers is 37 × 1, the maximum heat source intensity is 3.56 × 10¹⁰ W/m^3^. When the number of bundled fibers is 61 × 1, the maximum heat source intensity drops to 3.55 × 10¹⁰ W/m^3^, which is not significant. This shows that as the number of bundled fibers increases, the total heat source intensity of the optical fiber does not change significantly, reflecting the uniform distribution of the heat source inside the optical fiber.

**Figure 10:**
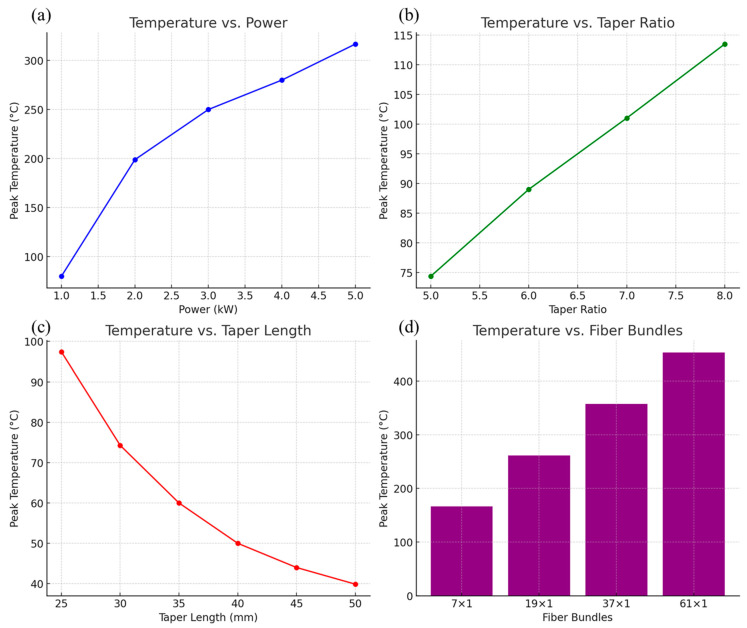
The influence of key parameters on the peak temperature of optical fiber. (**a**) As power increases from 1 kW to 5 kW, temperature increases non-linearly; (**b**) the larger the cone ratio, the higher the temperature peak, indicating that the thermal effect increases as the cone ratio increases; (**c**) increasing cone length helps reduce temperature peaks and disperse local heat; (**d**) as the number of bundled fibers increases from 7 × 1 to 61 × 1, the temperature peak increases significantly.

We have summarized the relationship between temperature and power, taper ratio, taper length, and number of bundled fibers, as shown in Figure 10.

Figure 10 shows the influence of input power, taper ratio, taper length and number of bundled fibers on the thermal effect of the fused-tapered fiber. As the input power increases, the temperature peak rises significantly, especially under high power conditions; heat accumulation intensifies, showing an obvious nonlinear growth trend; the increase in the cone ratio leads to a concentration of power density, further increasing the temperature peak in the cone area. This needs to be optimized in the design to balance optical performance and thermal stability; the extension of the taper length helps to disperse heat evenly, significantly reduces the temperature peak and improves heat distribution, thereby improving the thermal stability of the system; while the increase in the number of fiber bundles significantly increases the temperature peak, especially in high-density fiber bundles, where the local heat accumulation effect is particularly obvious.

The results demonstrate that power, taper ratio, taper length, and bundle configuration are critical factors influencing the thermal effects in fused-tapered optical fibers. Power levels and taper ratios dictate the extent of local heat accumulation, with higher values exacerbating temperature peaks. In contrast, extending the taper length effectively reduces localized overheating, promoting a more uniform thermal distribution. Bundling multiple fibers intensifies temperature gradients but does not significantly alter the heat source intensity, pointing to the role of inter-fiber heat conduction as a dominant factor in temperature rise.

Compared to previous studies, this analysis provides a deeper understanding of the interplay between geometric parameters and thermal behavior in optical fibers. These findings offer practical insights for designing high-power fiber laser systems, with implications for optimizing fiber geometry and implementing advanced cooling solutions to enhance system stability and performance.

## 4. Conclusions

This study investigates the thermal effects of fused-tapered passive optical fiber signal combiners, focusing on the influence of key parameters such as power, taper ratio, taper length, and fiber bundle configurations. By employing theoretical modeling and finite element simulations, we have identified critical factors affecting the thermal behavior of these systems. The main conclusions are as follows:

(1) The temperature gradient in tapered optical fibers and signal combiners is primarily axial, with the highest temperature at the tapered output end. Simulations reveal that heat is transferred from the output to the input, with the output end accumulating the most heat. For instance, in 61 × 1 fiber bundles, the peak temperature at the tapered output reaches 453.09 °C, while the input end remains much cooler, creating a distinct axial gradient.

(2) As power increases, the temperature of the optical fiber rises sharply, with a more uneven distribution and significant heat accumulation. At low power, the temperature is relatively uniform. However, as power increases from 1 kW to 5 kW, the peak temperature surges from 80 °C to 316.73 °C (296% increase), and the local temperature gradient at the tapered output triples. Heat source intensity also increases from 2.36 × 10¹⁰ W/m^3^ to 11.8 × 10¹⁰ W/m^3^ (400% increase), underlining the need for effective heat dissipation at high power levels.

(3) Increasing the tapering ratio raises both the temperature and heat source intensity, concentrating thermal effects at the tapered output. As the taper ratio increases from 5 to 8, the peak temperature rises by 52.5%, from 74.37 °C to 113.46 °C, and heat source intensity increases by 179%. A higher tapering ratio intensifies local heat accumulation, but reducing the ratio can significantly lower the temperature peak and improve thermal stability, with a 34.5% reduction in peak temperature when the ratio decreases from 8 to 5.

(4) Increasing the tapered length results in more uniform heat distribution and a reduced temperature peak. When the length increases from 25 mm to 50 mm, the peak temperature decreases by 59.1%, from 97.41 °C to 39.87 °C, and heat source intensity drops by 74.5%. A longer tapered length helps spread heat more evenly, reducing local overheating and enhancing the overall thermal stability of the fiber.

(5) Bundling multiple fibers significantly increases the temperature peak, while heat source intensity remains stable. For 7 × 1 bundles, the peak temperature is 166.78 °C, rising to 453.09 °C for 61 × 1 bundles (171.6% increase). However, the heat source intensity remains relatively unchanged, from 3.67 × 10¹⁰ W/m^3^ to 3.55 × 10¹⁰ W/m^3^ (3.3% decrease). The main effect of bundling is a concentration of heat at the tapered output end, where the temperature gradient increases by 167.4%. Optimizing fiber bundling and heat dissipation can reduce temperature peaks and improve the uniformity of heat distribution.

These findings provide a comprehensive understanding of the thermal dynamics in fused-tapered optical fiber signal combiners, offering valuable insights for optimizing their design and ensuring reliable operation under high-power conditions. Future research should focus on advanced thermal management techniques and material innovations to further enhance the performance and durability of high-power optical fiber systems.

## Figures and Tables

**Table 1:** Basic parameters.

Parameters	Numeric
Taper Ratio	10
Adiabatic taper length	20 mm
Laser input power	2 kW
Specific heat capacity c	703 J/kg·K
Thermal conductivity k	1.38 W/m·K
Convective heat transfer coefficient h	10 W/m^2^·K

**Table 2:** Simulated computational costs and parameter settings.

Simulation Group	Input Power (kW)	Taper Ratio	Taper Length (mm)	Number of Combined Optical Fibers	Single Calculation Time (Hours)	Total Calculation Times
1	1	10	20	Single root	2.5	3
2	2	10	20	Single root	2.5	3
3	3	10	20	Single root	2.5	3
4	4	10	20	Single root	2.5	3
5	5	10	20	Single root	2.5	3
6	2	5	20	Single root	2.8	3
7	2	6	20	Single root	2.8	3
8	2	7	20	Single root	2.8	3
9	2	8	20	Single root	2.8	3
10	2	10	25	Single root	3	3
11	2	10	30	Single root	3	3
12	2	10	35	Single root	3	3
13	2	10	40	Single root	3	3
14	2	10	45	Single root	3	3
15	2	10	50	Single root	3	3
16	2	10	20	7 × 1	3.5	3
17	2	10	20	19 × 1	3.8	3
18	2	10	20	37 × 1	4	3
19	2	10	20	61 × 1	4	3

**Table 3:** Relationship between casing and bundle root size.

Number of Beam Layers	Number of Roots	Number of Diagonals	Diagonal Length (mm)	Diagonal Length Casing Diameter Length (mm)
1	1	1	0.4	0.8
2	7	3	1.2	2.4
3	19	5	2	4
4	37	7	2.8	5.6
5	61	9	3.6	7.2

## Data Availability

Data are contained within the article.
